# Use of oral health care services by family caregivers and care recipients: the LENTO intervention

**DOI:** 10.2340/aos.v83.40687

**Published:** 2024-05-22

**Authors:** Shanza Razzaq, Irma Nykänen, Tarja Välimäki, Sohvi Koponen, Roosa-Maria Savela, Ursula Schwab, Anna Liisa Suominen

**Affiliations:** a Institute of Dentistry, University of Eastern Finland, Kuopio, Finland; b Kuopio Research Centre of Geriatric Care, School of Pharmacy, Faculty of Health Sciences, University of Eastern Finland, Kuopio, Finland; c Institute of Public Health and Clinical Nutrition, Faculty of Health Sciences, University of Eastern Finland, Kuopio, Finland; d Department of Nursing Science, Faculty of Health Sciences, University of Eastern Finland, Kuopio, Finland; e Department of Endocrinology and Clinical Nutrition, Kuopio University Hospital, Kuopio, Finland; f Oral Health Teaching Unit, Kuopio University Hospital, Kuopio, Finland

**Keywords:** Oral health care services, family caregivers, care recipients, oral health intervention

## Abstract

**Objective:**

We aimed to evaluate the impact of an individually tailored preventive oral health intervention on the use of oral health care services by older family caregivers (FCs) and their care recipients (CRs).

**Material and methods:**

A randomized controlled six and 12-month oral health intervention study included FCs and CRs aged ≥65 years living in Eastern Finland. The participants were randomly assigned to an intervention (FCs *n* = 53, CRs *n* = 47) and a control (FCs *n* = 39, CRs *n* = 35) group. Individually tailored oral health interventions for the FCs provided by a dental hygienist focused on oral hygiene and self-care. Generalized estimating equations were used to analyze the impact of intervention on the change in the use of oral health care services.

**Results:**

The intervention had no significant effect on the use of oral health care services by the FCs or their CRs. Traditional factors such as female gender, a higher number of teeth, toothache, no dental fear, and higher morbidity were significantly (*p* < 0.05) associated with an increased use of oral health care services in the FCs, but not among the CRs.

**Conclusions:**

Individually tailored preventive oral health intervention showed no effect on the use of oral health care services. To promote oral health among the elderly, specific interventions focusing on use of oral health care services are needed.

**Trial registration:**

clinicaltrials.gov/study/NCT04003493

## Introduction

Oral health is pertinent to general health and well-being, and also enhances the quality of life in older people [1,2]. Impaired oral health is common among ≥65-year-olds, including the loss of natural teeth, oral infections, periodontal diseases, dental caries, mucosal lesions, temporomandibular dysfunction, dry mouth, and oral cancer [3–5]. Research has demonstrated that oral diseases are also associated with chronic systemic diseases e.g., cardiovascular, cerebrovascular, and respiratory diseases, and diabetes [6,7]. These chronic diseases share a common risk factor with many oral diseases [8].

The global population is rapidly aging, especially in Finland, which has one of the oldest populations in Europe. In 2022, 22% of Finns were aged ≥65 years, and this will increase to 26% by 2030 and to 29% by 2060 [9]. Finnish municipalities look after the health and well-being of elderly people and encourage them to live in their homes until the end of their lives by providing support and care at home [10]. In 2022, there were 23,138 registered family caregivers (FCs) aged ≥65 years [11], who were more likely to be the spouses of the care recipients (CRs). These FCs play an important role in providing home care to their cognitively, medically, or functionally dependent CRs [12].

The regular use of oral health care services is a prerequisite for good oral health [13]. The burden of oral diseases increases with increasing age, causing a consequently increased need for preventive and operative oral health care [14]. Access to and the use of oral health care services are instrumental to disease prevention, health promotion, and the timely diagnosis, and treatment of oral diseases [15]. In older people, factors associated with the use of oral health care services include age, gender, the level of education, income, the general and oral health status, oral health awareness and attitudes, and the perceived need for dental care [16,17]. Factors that act as a barrier to accessing oral health care services include multimorbidity [18], functional dependence and cognitive impairment [19], poor access to care, and the lack of finances [20]. This has been shown in Finland too [21], where the oral health care system is comprised of a combination of private and public sectors and is funded by out-of-pocket payments and both tax-based and social insurance systems [22].

Interventions could aid in promoting the use of health care services, but research especially among the older population is scarce [23]. Most previous intervention studies among caregivers and CRs have focused on institutionalized or nursing home CRs and their caregivers [24]. One intervention study has focused on the use of oral health care services among home care clients aged ≥75 years [25]. To the best of our knowledge, no randomized controlled trials exist on individually tailored oral self-care guidance for older FCs and their CRs aged ≥65 years. The purpose of this study was to evaluate the impact of individually tailored preventive oral health intervention on the use of oral health care services in older FCs and their CRs.

## Methods

### Study design

This study employed data from the Lifestyle, Nutrition, and Oral Health in Caregivers (LENTO) intervention study on older FCs and their CRs living in an urban and a rural municipality in eastern Finland [26]. All FCs with a valid care allowance (CA) in the municipality registers of Kuopio and Vesanto between June and December 2019 and their CRs living at home were recruited. In Finland, municipalities grant a CA to FCs, which includes 3 days per month leave from work, taxable fees, services for the CR, and informal care support services. CA takes into consideration both the CRs’ and the FCs’ need for support and services. Support cases are usually handled by the (domestic services) case manager or a social worker for the elderly or the disabled. The criteria for granting support, their assessment methods and the amount of CA therefore vary from one municipality to another. This CA in participating municipalities Kuopio and Vesanto is granted to those FCs whose CRs cannot cope independently with everyday activities due to an illness, disability, or other special need for care. CRs receiving end-of-life treatment, along with their FCs, were not included in this study [27]. Those FCs whose CRs were aged ≥65 years, except for three who were less than 65 years of age but were in the FCs registers of older people because they had been diagnosed with Alzheimer’s disease, were contacted by the service manager for aged people through an invitation letter. The invitation letter had three components: a written invitation letter from the researchers, a written invitation letter from the city of Kuopio to take part in the study (only for Kuopio residents), and detailed information regarding the study provided by Kuopio University Hospital and the University of Eastern Finland.

The study participants (FCs together with their CRs) were randomized into intervention and control groups through a computer-generated allocation mechanism with an allocation ratio of 1:1. Randomization of the study participants was performed using IBM SPSS Statistics software (v. 27, IBM Corp., Armonk, NY). The nutritional intervention included individualized nutritional care provided to the FCs and the oral health intervention included verbal and written instructions about oral and denture hygiene provided to the FCs. Data were collected at three time points, namely at baseline and after 2 months (only in the intervention group), 6 months, and 12 months, with questionnaires, interviews, and clinical examinations, including oral health. Neither the researchers nor the participants were blinded because of the type of intervention on oral health and nutrition, i.e., individualized care and instructions were given by the researchers. The Research Ethics Committee of Northern Savo Hospital District, Finland, approved this study, and the trial was registered in the ClinicalTrials.gov database (NCT04003493, registered 1 July 2019). Written informed consent was received from all the participants.

### Study population

**Figure 1:**
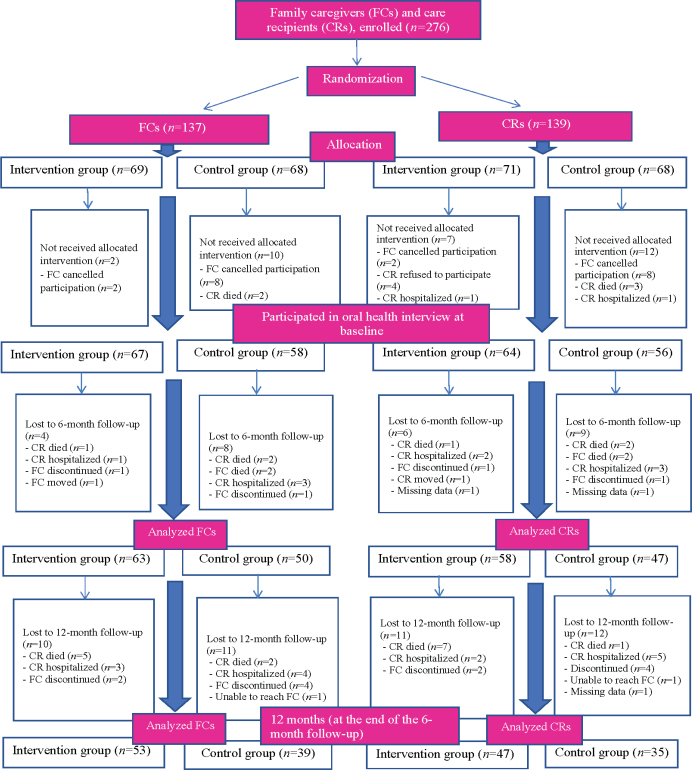
Flowchart of the study population who participated in interviews concerning oral health.

This study included those who participated in interviews about oral health in each three time points (Figure 1). At baseline, 125 FCs and 120 CRs living in the city of Kuopio and the municipality of Vesanto in 2019 took part in the oral health interviews. The sample size estimations of this study were based on the amount of plaque, with a difference of 20% in this amount between the control and intervention groups (power 0.80 and α 0.05). To detect a statistically significant difference between these two groups, a sample size of 120 (i.e., *n* = 60/group) was required.

### Data collection

The interviews and clinical examinations were planned to be carried out by a trained nurse and a dental hygienist during four home visits in the intervention group (at baseline and after 2 months, 6 months, and 12 months) and three home visits in the control group (at baseline and after 6 months, and 12 months). At baseline, a trained nurse first conducted a home visit, followed a week later by a home visit by a dental hygienist, who conducted oral health interviews and clinical examinations. For the intervention group, individually tailored oral self-care care instructions based on both the FCs’ and their CRs’ need were provided to the FCs by the dental hygienist during the home visits. The need of both the FCs and their CRs was determined on the basis of interviews and clinical examinations, and instructions given to FCs were assumed to be delivered also to their CRs. The oral health intervention contained at least one of the following sets of verbal and written instructions: dental hygiene instructions (tooth brushing, interdental cleaning), denture hygiene instructions (storing and cleaning of dentures), and cleaning and management of the oral mucosa and dry mouth. At 2 months, the dental hygienist performed a home visit for the intervention group, providing oral health care guidance only. At 6 months after baseline (between December 2019 and 16 March 2020), a dental hygienist conducted clinical oral health examinations and interviews during one home visit. Due to restrictions resulting from the COVID-19 pandemic (between 17 March 2020 and June 2020), some of the interviews [FCs *n* = 52 (46.0% of the FCs involved at this time point), CRs *n* = 44 (41.9% of the CRs involved at this time point)] were conducted by a dental hygienist by phone and no clinical examinations were conducted. At 12 months after baseline (between June 2020 and December 2020), the dental hygienist conducted a home visit. Due to the COVID-19 pandemic, the interview forms were sent to the participants by post before the visit and were carefully checked by the dental hygienist during the home visit, or participants were interviewed by the dental hygienist if they had not completed them. During the COVID-19 pandemic, necessary precautions and safety measures were taken during the home visits, and clinical examinations that were absolutely necessary were performed at the FCs’ homes, with their permission.

### Measurements

The oral health interviews of the FCs and CRs included questions regarding the use of and views on oral health care services, the perceived need for dental care, self-reported oral health, oral health-related behavior, oral health-related quality of life, and the assistance of CRs by caregivers in oral care. The dental hygienist carried out clinical examination of the participants in sitting or lying down position, with the help of a headlamp, mouth mirror and WHO periodontal probe [28].

The primary outcome in this study was use of oral health care services during the previous year reported by the participants at baseline, after the 6-month follow-up, and after the 12-month follow-up. This was enquired with the question, ‘When did you last time visit oral health care?’ with the response options (1) during the previous year, (2) 1–3 years ago, (3) 4–5 years ago, (4) over 5 years ago, and (5) I have never visited dental care. Those who choose option ‘1’ were defined as having visited oral health care during the previous year and the rest of response options were combined as having not visited oral health care.

### Associated factors

The interview questions included in this study and asked by the dental hygienist are given in Table 1. The socioeconomic and sociodemographic status (age, gender, education, income, marital status, municipality of residence), and general health information of the participants were collected by a trained nurse at baseline. The number of teeth were recorded during intraoral clinical examinations. The use of medication and supplements was based on medication lists and daily prescriptions [27]. Comorbidity was determined by using a modified version of the Functional Comorbidity Index (FCI) [29]. The activities of daily living (ADL) were assessed with the Barthel Index [30], and the instrumental activities of daily living (IADL) with the Lawton and Brody scale [31]. The cognitive status was determined using the Mini-Mental State Examination (MMSE) [32], symptoms of depression using the Geriatric Depression Scale (GDS-15) [33], and sense of coherence (SOC) using Antonovsky’s [34] Orientation to Life Questionnaire. The measurements of ADL, IADL, MMSE, GDS-15 and SOC were only performed for FCs.

**Table 1:** Interview questions used and asked by the dental hygienists.

QUESTION	ANSWER	VARIABLE USED IN ANALYSES
**PRIMARY OUTCOME** When did you last time visit oral health care?	1) During the previous year 2) 1–3 years ago 3) 4–5 years ago 4) Over 5 years ago 5) I have never visited dental care	Option ‘1’ was defined as having visited oral health care services during the previous year and the rest were combined as having not
**ASSOCIATED FACTORS** Do you have a habit of visiting a dentist?	1) Regularly for check-ups 2) Only when in pain or trouble 3) Never	Response ‘1’ was defined as undergoing regular check-ups and ‘2’ or ‘3’ having not
When you last visited oral health care, who did you visit during the treatment period? (Several answer options were allowed)	1) A dentist 2) A dental hygienist 3) A dental nurse 4) A dental technician	
Do you currently need dental care?	Yes or no	
How do you rate your oral health at the moment?	1) Good 2) Rather good 3) Moderate 4) Rather poor 5) Poor	Options ‘1’ and ‘2’ were together defined as good self-reported oral health and options ‘3 to 5’ were defined as average/poor oral health.
Have you during the past 12 months had toothache or other problems related to your teeth or dental prostheses?	1) No 2) Occasionally 3) Continuously	Response ‘1’ was categorized as having no toothache or oral discomfort and ‘2’ or ‘3’ as yes
How often do you brush your teeth?	1) More often than twice a day 2) Twice a day 3) Once a day 4) Less frequently 5) Never	Options ‘1’ or ‘2’ were defined as brushing at least twice a day and ‘3’ or ‘4’ or ‘5’ as having not
Denture status: Which of the following do you have?	1) Natural teeth, no dental prostheses 2) Partial dentures with natural teeth 3) Full prostheses (no natural teeth or tooth remnants 4) No dental prostheses or natural teeth	Options ‘2’ or ‘3’ were defined as using removable dentures and ‘1’ or ‘4’ as having not
Did the following factors prevent you from accessing the oral health services you wanted? (Several answer options were allowed)	1) Queueing to access care 2) Poor connections to the place of treatment 3) High cost of care 4) Dental fear 5) Inappropriate treatment 6) Other: _______	Those selected ‘4’ were considered as having dental fear.
What was the reason for visiting the oral health care services last time? (Several answer options were allowed)	1) Toothache 2) Some other pain or trouble related to face, mouth, teeth or dental prostheses 3) Injury 4) Appearance related factors/check-up 5) Recall 6) Appointment given at last treatment 7) Other: _______	Those selected ‘5’ were considered as having recalled and ‘4’ as having check-up

#### Statistical analysis

Statistical comparisons of differences in distributions were performed for categorical variables with the Pearson chi-squared test and for continuous variables with the Mann–Whitney *U-* test or independent samples *t-*test. Generalized estimating equations (GEE) with binary logistic regression were first used to analyze the difference in the age- and gender-adjusted change in the use of oral health care services over the 12-month intervention within the groups between the baseline and the 6- and 12-month measures, and between the intervention and control groups of FCs and CRs (Table 2). Multivariate analysis (GEE) was further used to analyze the impact of the intervention on the change in the use of oral health care services during the previous year, adjusted for age, gender, socioeconomic status, oral health, dental fear, recall, number of teeth, functional ability (IADL), morbidity (FCI), cognition (MMSE), and depression (GDS-15). GEE models were run separately for the FCs and CRs. A *p*-value of less than 0.05 was considered statistically significant. Data analyses were performed using IBM SPSS Statistics software (v. 27, IBM Corp., Armonk, NY).

**Table 2:** Baseline characteristics of the family caregivers (*n* = 125) and the care recipients (*n* = 120).

CAREGIVERS, total n = 125	Intervention group, *n* = 67				Control group, *n* = 58				*P^a^*
	Mean	SD	*n*	%	Mean	SD	*n*	%	
**Demographic and economic characteristics**									
Age	74.7	6.5			74.4	8.1			0.817^c^
Females			47	70.1			42	72.4	0.780
Education in years^d^	11.1	3.8			11.1	3.3			0.713^b^
Household income (euros)	3122.0	1012.0			3170.7	795.2			0.561^b^
**Oral health-related characteristics**									
Regular use of oral health care services			18	26.9			8	13.8	0.175
Use of oral health care services during the previous year			50	74.6			35	60.3	0.066
Supplier of care during the previous treatment period: Dentist			53	79.1			45	77.6	0.837
Dental hygienist^e^			26	39.4			20	34.5	0.572
Dental nurse^e^			8	12.1			7	12.1	0.993
Dental technician^e^			9	13.6			6	10.3	0.575
Perceived need for dental care^e^			25	37.9			21	36.2	0.848
Good self-reported oral health^e^			42	63.7			34	58.6	0.757
Tooth brushing frequency at least twice daily^f^			43	71.7			43	84.3	0.397
Wearing a removable prosthesis			34	50.7			24	41.4	0.439
**Health-related characteristics**									
FCI	2.3	1.7			2.1	1.4			0.636^b^
Number of drugs in regular use^e^	5.6	4.0			5.0	3.2			0.355^b^
ADL^e^	97.8	3.4			98.2	3.7			0.574^b^
IADL	7.7	0.6			7.9	0.5			0.056^b^
SOC-13^e^	62.2	6.4			61.2	7.1			0.354^b^
MMSE^e^	26.0	3.2			26.8	2.8			0.418^b^
GDS-15^e^	3.1	2.3			2.9	2.6			0.501^b^
CARE RECIPIENTS, total, *n* = 120	Intervention group, *n* = 64				Control group, *n* = 56				*P^a^*
	Mean	SD	*n*	%	Mean	SD	*n*	%	
**Demographic characteristics**									
Age	79.6	7.8			79.1	7.7			0.733^c^
Females			24	37.5			18	32.1	0.539
**Oral health-related characteristics**									
Regular use of oral health care services			14	21.9			13	23.2	0.631
Use of oral health care services during the previous year			31	48.4			24	42.9	0.649
Supplier of care during the previous treatment period: Dentist			47	73.4			40	71.4	0.806
Dental hygienist			24	37.5			14	25.0	0.142
Dental nurse			6	9.4			4	7.1	0.659
Dental technician			13	20.3			8	14.3	0.386
Perceived need for dental care^g^			30	47.6			23	41.1	0.465
Good self-reported oral health^h^			22	35.5			27	49.1	0.690
Tooth brushing frequency at least twice daily^f^			20	39.2			22	50.0	0.507
Wearing a removable prosthesis^i^			35	54.7			28	50.9	0.730
**Health-related characteristics**									
FCI^j^	3.2	2.1			3.3	2.0			0.796^b^
Number of drugs in regular use^i^	8.8	4.0			8.8	4.3			0.843^b^

SD: standard deviation; FCI: Functional Comorbidity Index (a higher sum score indicates greater comorbidity); GDS-15: Geriatric Depression Scale (0–15, a higher score indicates severe depression); ADL: Barthel Index (a higher score, 91–99, indicates slight dependency); IADL: Instrumental activities of daily living (scale 0–8, a higher score indicates better functioning); SOC-13: Sense of coherence (13–91 points, a higher score indicates a stronger sense of coherence); MMSE: Mini-Mental State Examination (scale 0–30, a higher score indicates mild cognitive impairment), drugs in regular use (10 drugs per day is excessive polypharmacy).

a Difference between groups with Pearson’s chi-squared test.

b Difference between groups with the Mann–Whitney *U-* test (non-normally distributed outcomes).

c Difference between groups with the independent samples *t*-test (normally distributed outcomes).

d Intervention group FCs *n* = 66, control group FCs *n* = 57.

e Intervention group FCs *n* = 66.

f Intervention group FCs *n* = 60, CRs *n* = 51, control group FCs *n* = 51, CRs *n* = 44.

g Intervention group CRs *n* = 63.

h Intervention group CRs *n* = 62, control group CRs *n* = 55.

i Control group CRs *n* = 55.

j Intervention group CRs *n* = 63, control group CRs *n* = 53.

## Results

At baseline, the overall mean age of the FCs was 74.6 ± 7.3, 71% were females, 37% reported a need for dental care, 61% had good self-reported oral health, and 21% regularly visited a dentist for check-ups. Of the FCs, 68% had visited oral health care within the previous year. During their last treatment period, 78% went to a dentist and 37% visited a dental hygienist. The most common reason for the last visit to oral health care was an oral check-up (41%), while the second most common reason was being recalled (23%). As a barrier to oral health care, 53% of the FCs reported the waiting list, whereas 28% reported the high cost of dental treatment.

Similarly, the overall mean age of the CRs was 79.4 ± 7.7, 35% were females, 44% reported a need for dental care, 42% had good self-perceived oral health, and 22% regularly visited a dentist for check-ups. Of the CRs, 46% had visited oral health care within the previous year. During their last treatment period, 72% went to a dentist and 32% went to a dental hygienist. The most common reason for the last visit to oral health care was an oral check-up (36%), while the second most common reason was being recalled (24%). As a barrier to oral health care, 44% of the CRs reported the waiting list, whereas 19% reported the high cost of dental treatment.

According to the baseline characteristics, there were no statistically significant differences between intervention and control groups (Table 2). The difference between the intervention and control groups of FCs in IADL approached statistical significance (*p* = 0.056), as did the use of oral health care services during the previous year, being 74% in the intervention group and 60% in the control group (*p* = 0.066).

The use of oral health care services during the previous year increased statistically significantly only in the intervention group of CRs (*p* < 0.001) (Table 3). The difference between the intervention and control groups of CRs in the change in use of oral health care services was almost significant (*p* = 0.058). A statistically significant decrease in visits to a dentist was observed in both the intervention and control groups of FCs (*p* = 0.001 and *p* < 0.001, respectively) and CRs (*p* = 0.001 and *p* = 0.002, respectively). The change in the proportion of those who reported a check-up as the reason for the last visit was almost significantly different between the intervention and control groups of FCs (*p* = 0.049). There were no significant differences within the groups in the percentage of those with a good self-reported oral health status. However, the change in good self-reported oral health was significantly larger in the intervention groups of both the FCs (*p* < 0.001) and CRs (*p* = 0.020) than in their control groups.

**Table 3:** Changes in the use of oral health care services, the perceived need for dental care, and good self-reported oral health in the intervention and control groups of family caregivers and care recipients.

CAREGIVERS	Intervention group							Control group							*P* ^b^
	Baseline *n* = 67		6 months *n* = 63		12 months *n* = 53		*P^a^*	Baseline *n* = 58		6 months *n* = 50		12 months *n* = 39		*P* ^a^	
	*n*	%	*n*	%	*n*	%		*n*	%	*n*	%	*n*	%		
Use of oral health care services during the previous year	50	74.6	46	73.0	39	73.6	0.834	35	60.3	35	70.0	26	66.7	0.382	0.215
Supplier of care: Dentist	53	79.1	43	64.2	38	56.7	**0.001**	45	77.6	36	63.2	31	53.4	**< 0.001**	0.766
Dental hygienist	26	39.4	21	31.3	24	35.8	0.699	20	34.5	9	15.8	13	22.4	0.069	
Perceived need for dental care	25	37.9	25	39.7	19	35.8	0.809	21	36.2	14	28.0	16	41.0	0.659	0.590
Good self-reported oral health	42	63.7	43	68.2	35	66.0	0.933	34	58.6	33	66.0	33	59.0	0.318	**<0.001**
Reasons for previous visit: Check-up	31	47.7	29	46.0	22	41.5	0.442	20	35.1	14	28.0	14	35.9	0.964	**0.049**
Recall	12	18.8	14	22.6	8	15.1	0.556	16	28.1	11	22.0	9	23.1	0.484	0.373
Barriers to oral care access: Waiting list	13	54.2	11	52.4	12	60.0	0.641	17	53.1	6	42.9	15	71.4	0.192	0.812
High cost	4	16.7	4	19.0	5	25.0	0.482	12	37.5	4	26.7	6	28.6	0.542	0.150
CARE RECIPIENTS	Intervention group							Control group							*P* ^b^
	Baseline *n* = 64		6 months *n* = 58		12 months *n* = 47		*P^a^*	Baseline *n* = 56		6 months *n* = 47		12 months *n* = 36		*P* ^a^	
	*n*	%	*n*	%	*n*	%		*n*	%	*n*	%	*n*	%	
Use of oral health care services during the previous year	31	48.4	37	63.8	36	76.6	**<0.001**	24	42.9	23	48.9	18	50.0	0.359	**0.058**
Supplier of care: Dentist	47	73.4	39	60.9	32	50.0	**0.001**	40	71.4	36	66.7	26	48.1	**0.002**	0.965
Dental hygienist	24	37.5	14	21.9	16	25.0	**0.052**	14	25.0	8	14.8	10	18.5	0.122	0.117
Perceived need for dental care	30	47.6	26	44.8	27	57.4	0.207	23	41.1	17	37.0	12	33.3	0.393	0.110
Good self-reported oral health	22	35.5	22	38.0	17	36.2	0.554	27	49.1	21	45.7	19	52.7	0.246	**0.020**
Reasons for previous visit: Check-up	25	39.7	21	36.2	15	32.9	0.337	16	30.8	17	37.8	10	28.6	0.817	0.565
Recall	15	23.8	8	13.8	10	21.3	0.744	12	23.5	11	24.4	5	14.3	0.232	0.912
Barriers to oral care access: Waiting list	9	47.4	7	35.0	10	55.6	0.927	9	40.9	5	29.4	7	35.0	0.654	0.261
High cost	6	31.6	5	25.0	4	21.1	0.686	2	9.1	3	17.6	3	13.6		0.185

a Difference between 0-, 6-, and 12-month measures according to generalized estimating equations adjusted for age and gender.

b Difference between groups according to generalized estimating equations adjusted for age and gender.

(Statistically significant *p*-values in bold).

Among the FCs (Table 4), a higher number of teeth and comorbidity increased the probability of having visited oral health care services during the previous year (OR: 1.3, 95% CI: 1.1–1.5, and OR: 1.7, 95% CI: 1.0–2.8, respectively). Those FCs who were males (OR: 0.3, 95% CI: 0.1–1.0), who did not experience toothache or other problems related to their teeth or dentures during previous year (OR: 0.08, 95% CI: 0.01–0.6), who had dental fear (OR: 0.04, 95% CI: 0.003–0.5), and those who had depressive symptoms (OR: 0.8, 95% CI: 0.6–1.0) had lower odds of an increased use of oral health care services. Among the CRs, none of the baseline characteristics were associated with the change in the use of oral health care services during the previous year (Table 5).

**Table 4:** Association of baseline characteristics with the increase in the use of oral health care services during the previous year among family caregivers (FCs, *n* = 122).

Baseline characteristics	OR	95% CI	*P*
Intervention group (ref. control)	1.5	0.5–4.6	0.525
**Age (years)**	**1.1**	**1.0**–**1.2**	**0.065**
**Males (ref. females)**	**0.3**	**0.1**–**1.0**	**0.043**
Education (years)	0.9	0.7–1.1	0.196
Income	1.0	1.0–1.0	0.934
**Number of teeth**	**1.3**	**1.1**–**1.5**	**0.001**
No perceived need for dental care (ref. yes)	2.2	0.6–8.1	0.221
Good self-reported oral health (ref. poor)	1.9	0.5–6.5	0.323
Recalled (ref. no)	1.8	0.3–9.4	0.495
**No toothache or other problems related to dentures (ref. continuously)**	**0.08**	**0.01**–**0.6**	**0.017**
**Dental fear (ref. no)**	**0.04**	**0.003**–**0.5**	**0.016**
No removable prosthesis (ref. yes)	0.2	0.03–1.4	0.105
IADL	1.8	0.8–4.3	0.173
**FCI**	**1.7**	**1.0**–**2.8**	**0.025**
MMSE	0.9	0.8–1.1	0.438
**GDS-15**	**0.8**	**0.6**–**1.0**	**0.058**
1^st^ time for measurement (ref. 3^rd^)	1.3	0.5–3.2	0.627
2^nd^ time for measurement (ref. 3^rd^)	1.4	0.7–3.2	0.353

Analyzed using generalized estimating equations adjusted for age, gender, socioeconomic status, oral health, dental fear, recall, number of teeth, functional ability (IADL), morbidity (FCI), cognition (MMSE), and depression (GDS-15).

FC: Family caregiver; OR: Odds ratio; 95% CI: 95% Confidence interval (statistically significant values in bold); IADL: Instrumental activities of daily living; FCI: Functional Comorbidity Index; MMSE: Mini-Mental State Examination; GDS-15: Geriatric Depression Scale.

**Table 5:** Association of baseline characteristics with the increase in the use of oral health care services during the previous year among care recipients (CRs, *n* = 115).

Baseline characteristics	OR	95% CI	*P* value
Intervention group (ref. control)	4.0	0.8–19.8	0.087
Age (years)	1.0	0.9–1.1	0.871
Males (ref. females)	0.4	0.1–1.7	0.230
Number of teeth	1.1	1.0–1.3	0.060
Good self-reported oral health (ref. poor)	3.0	0.2–37.1	0.400
Recalled (ref. no)	0.7	0.09–4.7	0.683
No toothache or other problems related to dentures (ref. continuously)	0.6	0.05–7.4	0.696
Dental fear (ref. no)	2.8	0.2–48.1	0.476
No removable prosthesis (ref. yes)	0.4	0.06–2.0	0.249
Functional Comorbidity Index	0.8	0.6–1.1	0.190
1^st^ time for measurement (ref. 3^rd^)	0.7	0.2–2.9	0.615
2^nd^ time for measurement (ref. 3^rd^)	0.7	0.2–2.4	0.610

Analyzed using generalized estimating equations adjusted for age, gender, socioeconomic status, oral health, recall, number of teeth, morbidity (FCI).

CR: Care recipient; OR: Odds ratio; 95% CI: 95% Confidence interval.

## Discussion

This study demonstrated that a preventive oral health intervention focusing on oral hygiene and self-care had no significant effect on the use of oral health care services by either FCs or CRs living in two municipalities of eastern Finland. Traditional factors such as female gender, a higher number of teeth, toothache, no dental fear, and higher morbidity were associated with an increased use of oral health care services in the FCs. This was not seen among the CRs, although their use of oral health care services slightly increased during the study in the intervention group.

One reason for not seeing the expected benefits of the intervention regarding the use of oral health care services in the FCs and CRs, in addition to the fact that the intervention did not directly focus on the use of such services, could have been the COVID-19 pandemic, which was in its early phase at the time of the LENTO intervention. In Finland, the government declared a state of emergency from mid-March to mid-June 2020 during the first wave of the COVID-19 pandemic, and implemented various restrictions and recommendations, including social distancing, the closure of public institutions, and advice to ≥70-year-olds to stay at home [35]. In our study, the mean age of participants was 74.6 ± 7.3 years for the FCs and 79.4 ± 7.7 years for the CRs, which meant that many participants belonged to the age group that was recommended to stay indoors, thus limiting access to and use of any health care services including oral health care. Even the alternative option of digital and telemedicine was unable to compensate for this decrease [35] which was seen globally [36]. A study assessing the adverse impact of the COVID-19 pandemic on diabetic patients in the North Karelia region of Finland, demonstrated that dental visits to public health services significantly decreased in these patients during the early phase of the pandemic [37]. However, similar results were obtained from another Finnish preventive intervention study targeted at older home care clients and conducted before the COVID-19 pandemic. Intervention had no significant effect on the use of oral health care services [25], indicating other probable reasons explaining our results. Considering the age group of the participants, a decline in general health during the 12 months of intervention may also be expected, which can potentially decrease the use of oral health care services.

Two-thirds of the FCs had already visited oral health care services in the previous year at baseline. This can be regarded as a high proportion and probably another reason for not seeing any effect of the intervention. The FCs having participated in our study were probably the most health-conscious, explaining their higher use of oral health care services. Also, 71% of the FCs were females, and previous studies have reported that the use of oral health care services was greater among women than men [21] and also among those women aged ≥70 years [38]. In addition to the female gender, the higher number of teeth, toothache, no dental fear, depression and higher morbidity were associated with increased use of oral health care services during the previous year among the FCs, but not among the CRs. To sustain the high level of use of services, we need to keep in mind these factors that have also previously been shown to be associated with the use of oral health care services among older Finnish adults [21,25,39–42]. The health and well-being of FCs is important to keep in focus, as they might neglect their own health while taking care of their CRs. A previous study on caregivers looking after CRs with Alzheimer’s disease revealed that they were less willing to use supportive services for themselves, as they did not want to leave their CRs under the supervision of other caregivers [43].

We observed that the proportion of CRs who had visited oral health care services during the previous year was lower than FCs in our study or on average among Finnish adults aged ≥65 years in 2011 [21]. This discrepancy could be explained by the difference between the study samples. The CRs in our sample had poor health and were dependent on their FCs, whereas the nationally representative Health 2011 Survey included healthy participants. However, the use of oral health care services during the previous year among CRs in our study (48% in the intervention group and 43% in the control group) was similar to older home care clients who had participated in a preventive intervention study (46% and 39% respectively) [25]. Experiences with various oral health care professionals (visits to dentist, dental hygienist, or dental technician) may not be the reason for the difference in visit rates between the FCs and their CRs since they were similar. We also observed that none of the traditional baseline characteristics studied were associated with the use of oral health care services during the previous year in the CRs contrary to previous studies on the use of oral health care services in older adults [19,21]. This may be due to the fact that older Finnish adults are in general less likely to use oral health care services compared to their counterparts for example in other Nordic countries because of higher number of edentulous older people in Finland [41]. A higher number of edentulous persons were observed among the CRs (20%) compared to the FCs (11%) which could be an explanation for CRs’ lower use of oral health care services. Holmavuo et al. [44] using this same data found that factors such as favorable perceptions of oral care were positively associated with the use of oral health care services during the previous year in the FCs, but not among the CRs. To improve equality in the use of oral health care services also among the older people with the poorest functional ability and cognitive impairment like the CRs, they may need home visits formulated as part of the oral health care regime of CRs as suggested by Komulainen et al. [19].

The strengths of our study were the randomized, population-based design, i.e., the target population comprised non-institutionalized CRs and their FCs, and the use of validated methods. One example of a validated measure was the use of a commonly applied indicator, ‘visits during the previous year’. The data were collected via semi-structured interviews conducted through home visits and phone calls, facilitating, and improving the participation of FCs. The study participants belonged to one specific region of Finland, i.e., North Savo in eastern Finland, which may be considered to limit the generalizability of our results. However, oral health care services are relatively uniform throughout Finland and regional differences should be quite small. The follow-up time of our study was 12 months, a long enough timeframe to observe changes in oral health behavior. The COVID-19 pandemic could have potentially affected the use of oral health care services among older people during this intervention and hence diluted some of the positive effects of the intervention. Blinding was impossible considering the design of the study i.e., intervention on oral health and nutrition, which may have caused performance bias. The small dataset of older FCs and their CRs, as well as their higher dropout rates (32.1% in FCs and 40.3% in CRs after 12-months of intervention) mainly due to death or hospitalization, or attrition especially due to COVID-19 pandemic, is a limitation of this study. The clinical oral examiners were trained and calibrated, but no repeated measures were conducted thus not allowing any intra-examiner and/or inter-examiner analyses.

Although the intervention had no significant effect on the use of oral health care services, a slight increase in use over the intervention was detected among the CRs. Hence, participation in the study, including clinical oral health examinations and inquiring about oral health, may have had a positive effect on the use of oral health care services. The increase in life expectancy will increase the proportion of old people living at home and needing support and care in the future. The regular use of oral health care services in such older people should be prioritized and ensured by policymakers and oral health professionals with the goal of improving and optimizing their oral health. Since individually tailored preventive oral health intervention focusing on oral hygiene and self-care showed no effect on the use of oral health care services, it is important to conduct specific interventions focusing on the use of oral health care services to improve oral health of the elderly.
